# A pilot study on the immunogenicity of dendritic cell vaccination during adjuvant oxaliplatin/capecitabine chemotherapy in colon cancer patients

**DOI:** 10.1038/sj.bjc.6605935

**Published:** 2010-10-05

**Authors:** W J Lesterhuis, I J M de Vries, E A Aarntzen, A de Boer, N M Scharenborg, M van de Rakt, D-J van Spronsen, F W Preijers, C G Figdor, G J Adema, C J A Punt

**Affiliations:** 1 Department of Medical Oncology, Radboud University Nijmegen Medical Centre, PO Box 9101, Nijmegen 6500 HB, The Netherlands; 2 Department of Tumor Immunology, Nijmegen Centre for Molecular Life Sciences, Radboud University Nijmegen Medical Centre, PO Box 9101, Nijmegen 6500 HB, The Netherlands; 3 Department of Internal Medicine, Canisius Wilhelmina Hospital Nijmegen, PO Box 9015, Nijmegen 6500 GS, The Netherlands; 4 Laboratory of Hematology, Department of Laboratory Medicine, Radboud University Nijmegen Medical Centre, PO Box 9101, Nijmegen 6500 HB, The Netherlands

**Keywords:** colon cancer, chemotherapy, dendritic cells, immunotherapy, oxaliplatin, vaccination

## Abstract

**Background:**

Dendritic cell (DC) vaccination has been shown to induce anti-tumour immune responses in cancer patients, but so far its clinical efficacy is limited. Recent evidence supports an immunogenic effect of cytotoxic chemotherapy. Pre-clinical data indicate that the combination of chemotherapy and immunotherapy may result in an enhanced anti-cancer activity. Most studies have focused on the immunogenic aspect of chemotherapy-induced cell death, but only few studies have investigated the effect of chemotherapeutic agents on the effector lymphocytes of the immune system.

**Methods:**

Here we investigated the effect of treatment with oxaliplatin and capecitabine on non-specific and specific DC vaccine-induced adaptive immune responses. Stage III colon cancer patients receiving standard adjuvant oxaliplatin/capecitabine chemotherapy were vaccinated at the same time with keyhole limpet haemocyanin (KLH) and carcinoembryonic antigen (CEA)-peptide pulsed DCs.

**Results:**

In 4 out of 7 patients, functional CEA-specific T-cell responses were found at delayed type hypersensitivity (DTH) skin testing. In addition, we observed an enhanced non-specific T-cell reactivity upon oxaliplatin administration. KLH-specific T-cell responses remained unaffected by the chemotherapy, whereas B-cell responses were diminished.

**Conclusion:**

The results strongly support further testing of the combined use of specific anti-tumour vaccination with oxaliplatin-based chemotherapy.

Until recently, the commonly held opinion was that chemotherapy and immunotherapy should not be combined because of the myelosuppressive effect of most cytotoxic agents. However, it has now become evident that chemotherapeutics can exhibit several beneficial effects on the immune system (Lake and Robinson, 2005). For example, treatment with gemcitabine results in increased antigen cross-presentation, T lymphocyte expansion and T-cell infiltration of tumours (Nowak *et al*, 2002), and 5-fluorouracil (5-FU) has been described to upregulate tumour antigen expression on colorectal and breast cancer cells (Correale *et al*, 2003). Furthermore, suppressive regulatory T cells are depleted by several chemotherapeutics also resulting in enhanced T-cell reactivity (Correale *et al*, 2005a; Ghiringhelli *et al*, 2007). In addition, treatment with non-myeloablative lymphodepleting chemotherapy results not only in reduced regulatory T-cell frequencies, but also provides ‘space’ in the bone marrow for naive T cells to proliferate upon adoptive T-cell transfusion (Dudley *et al*, 2002). Using this strategy, significant clinical results have been obtained in metastatic melanoma patients (Dudley *et al*, 2005). Pioneering work by Zitvogel and colleagues has shown that the cytotoxic agents oxaliplatin and doxorubicin induce immunogenic cell death, as upon treatment with these agents, tumour cells transport calreticulin to their cell surface. The exposure of calreticulin provides a signal that is recognised by dendritic cells (DCs) and ultimately results in phagocytosis of the tumour cells (Obeid *et al*, 2007). Exposure of tumour cells to oxaliplatin results in the release of high mobility group box 1 (HMGB1) protein, that activates DCs in a toll-like receptor-4 (TLR4)-dependent manner (Apetoh *et al*, 2007). The clinical impact of this immune pathway in colorectal cancer patients is emphasised by the recent observation that patients carrying the TLR4 loss-of function allele Asp299Gly exhibit reduced progression-free and overall survival in response to oxaliplatin as compared with patients carrying the normal TLR4 allele (Tesniere *et al*, 2009).

Previous studies in animal models provide a rationale for the combination of chemotherapy and immunotherapy in colon cancer. In a murine model, T-cell responses against the colon cancer-associated antigen thymidylate synthase that were induced by a peptide vaccine were not hampered by the administration of 5-FU. The combination of peptide vaccination and 5-FU resulted in a significant delay in tumour growth as compared with treatment with peptide or 5-FU alone (Correale *et al*, 2005b). Similarly, it was shown in a murine colon cancer model that the addition of 5-FU/leucovorin or irinotecan to bone marrow-derived DC vaccination did not diminish the immunogenicity of the vaccine (Bourquin *et al*, 2006). Small proof-of-concept clinical trials in cancer patients indicate that the efficacy of anti-cancer vaccines may indeed be enhanced by chemotherapy (Kaufman *et al*, 2008; Nistico *et al*, 2008), however additional studies on scheduling and appropriate combinations are warranted.

Platinum-based chemotherapy represents a cornerstone in the systemic treatment of many types of cancer (Desoize and Madoulet, 2002). Besides their direct cytotoxic effects, platinum anti-cancer drugs may also exert their clinical effect through indirect activation of the immune system via induction of immunogenic tumour cell death (Apetoh *et al*, 2007; Obeid *et al*, 2007; Tesniere *et al*, 2009). Most studies however, mainly focused on the effect of chemotherapy on tumour cells and antigen-presenting cells. Little attention has been paid to the effect of chemotherapy on the effector lymphocytes of the immune system. Although neutropenia is a common side effect of chemotherapy in the treatment of solid tumours, lymphopenia is rarely observed. A stimulatory effect of chemotherapy on tumour immunogenicity without impairing immune effector cell function would provide a strong rationale to develop novel chemoimmunotherapeutic strategies. For this reason, we investigated whether an oxaliplatin-based chemotherapy regimen combined with antigen-specific vaccination in cancer patients can result in tumour antigen-specific immune reactivity.

## Patients and methods

### Study design

This was an open-label, single-institution, single-arm exploratory study in which patients with stage III colon cancer received adjuvant treatment with monocyte-derived mature DCs loaded with carcinoembryonic antigen (CEA)-peptide in combination with standard oxaliplatin/capecitabine chemotherapy. Approval from the local regulatory committee was obtained.

### Objectives

The primary end point was to assess the immunogenicity of the vaccine during oaxaliplatin/capecitabine chemotherapy. Secondary end points were the toxicity and the feasibility of CEA-specific vaccination in colon cancer patients, during chemotherapy.

### Patients

Inclusion criteria included: patients with stage III colon cancer, HLA-A0201 phenotype, ECOG performance status 0–1, age above 18 years, initiation of adjuvant chemotherapy <8 weeks since surgery for the primary tumor, no previous chemotherapy, adequate bone marrow, kidney and hepatic function and written informed consent. Exclusion criteria included: the use of immunosuppressive drugs, a history of second malignancy and other serious concomitant diseases preventing the safe administration of study drugs or likely to interfere with the study end points.

### DC preparation

DCs were generated as described previously (de Vries *et al*, 2005b; Lesterhuis *et al*, 2006). Peripheral blood mononuclear cells (PBMC) were isolated by PureCell (Medicult, Jyllinge, Denmark) density gradient centrifugation (30 min, 4°C, 2100 r.p.m.), adherent monocytes were cultured in Cellgro medium enriched with 500 U ml^−1^ interleukin-4 and 800 U ml^−1^ granulocyte macrophage colony stimulating factor ( all CellGenix, Freiburg, Germany). KLH (10 *μ*g ml^−1^, Calbiochem, San Diego, CA, USA) was added at day 3 of culture and 2 days before harvesting, we added the maturation cocktail (prostaglandin E_2_ (Pharmacia & Upjohn, Puurs, Belgium, 10 *μ*g ml^−1^), tumor necrosis factor-*α* (10 ng ml^−1^), IL-1-*β* (5 g ml^−1^) and IL-6 (15 ng ml^−1^, all CellGenix)).

Cells were harvested at day 7 and part of the cells were loaded with peptide and put in a syringe for immediate vaccination; the remaining cells were frozen for the second and third vaccination and the delayed type hypersensitivity (DTH) (de Vries *et al*, 2002). Of each batch of patient DCs, a sample was used for quality control. Release criteria were as previously described (Figdor *et al*, 2004).

DCs were pulsed with the wild type CEA-peptide CAP-1 (CEA_571−579_, YLSGANLNL, Clinalfa, Bubendorf, Switzerland) (Tsang *et al*, 1995) directly after harvesting or after thawing (de Vries *et al*, 2002, 2003).

### Treatment schedule

**Figure 1:**
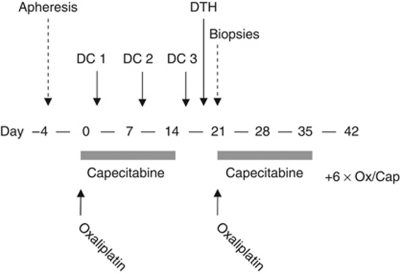
Treatment schedule.

Patients received eight cycles of oral capecitabine 2000 mg m^−2^ at days 1–14 and oxaliplatin 130 mg m^−2^ intravenously at day 1. Cycles were repeated every 3 weeks. Before the first cycle, patients underwent a leukapheresis for collection of PBMCs for the DC culture. At days 4, 10 and 17 of the first cycle of chemotherapy patients received three vaccinations intradermally (i.d.) and intravenously (i.v.; 5 × 106 i.d./10 × 106 i.v.) with CEA-peptide loaded dendritic cells (Figure 1). In pre-clinical models it has been shown that i.v. administered DCs provide a better anti-tumour response against visceral metastases, whereas non-visceral metastases respond better to i.d. administered DCs (Mullins *et al*, 2003). As both lymphogenous and haematogenous spreading occurs in colorectal cancer, we chose to combine both routes of administration. After completion of the vaccinations a DTH skin test was performed on day 19, followed by biopsies of DTH injection sites on day 22. Dexamethasone was not administered as anti-emetic prophylaxis during the first cycle.

### Immunologic monitoring

#### Analysis of peripheral blood immune cell subsets

Within the CD45+ leukocyte population, PBMCs were stained with antibodies against CD14, CD19, CD3, CD8, CD4 and CD56 (for distinction of monocytes, B cells, T cells, CTLs, T helper lymphocytes and NK cells, respectively). After washing, the samples were analysed by flowcytometry.

#### Monitoring of non-specific T-cell stimulatory capacity

Peripheral blood mononuclear cells were obtained by density-gradient centrifugation at days 1, 4, 10, 17 and 22. Cells were plated in 96-well U-bottom plates, 2 × 10^5^ per well, in RPMI/human serum of 5% and phytohaemagglutinin (PHA) was added (1 *μ*g ml^−1^). After 24 h, supernatant was harvested for cytokine production analysis. After 3 days, 1 *μ*Ci per well of 3H-thymidine was added to the culture for 8 h, after which proliferation was stopped by storing the culture plate at −20°C. Incorporation of 3H-thymidine was measured in a *β*-counter. The index was calculated as the counts ratio between PHA-stimulated PBMC and non-stimulated PBMC.

#### Monitoring of KLH-specific CD4+ T-cell responses

CD4+ T-cell responses against KLH were measured using a 3H-thymidine incorporation proliferation assay with PBMCs of the patients at days 1, 4, 10, 17 and 22. The index was calculated as the counts ratio between KLH-stimulated PBMCs and non-stimulated PBMCs.

#### Delayed type hypersensitivity reactions

Post-treatment DTH reactions were performed as described previously (de Vries *et al*, 2005a). Briefly, CEA-peptide alone (100 *μ*g in 100 *μ*l), DCs pulsed with CEA-peptide, DCs pulsed with KLH and CEA-peptide and DCs pulsed with KLH alone (0.4–5 × 10^5^ DCs each in ∼100–200 *μ*l) were injected i.d., 5–10 cm from an inguinal lymph node at different sites, in the back of the patients. The maximum diameter of induration was measured after 48 h. T-cell culture from DTH biopsies was performed in low dose IL-2 (Proleukin, Chiron, the Netherlands 100 U ml^−1^) for ∼2 weeks without *ex vivo* re-stimulation with antigen as described before (de Vries *et al*, 2005a).

#### MHC tetramer staining

DTH-derived cells (1 × 10^5^ cells in 10 *μ*l) or PBMCs (1 × 10^6^ cells in 10 *μ*l) were incubated with PE-labeled CEA and cytomegalovirus (CMV) tetrameric–MHC complexes (Sanquin, Amsterdam, the Netherlands) for 60 min at room temperature. In the last 20 min of this incubation, FITC-conjugated monoclonal antibodies directed against CD8 (Becton Dickinson, Breda, Netherlands) were added. After washing, the samples were analysed by flowcytometry. For peripheral blood at least 1 × 10^6^ PBMCs were analysed and for DTH-infiltrating T cells all available cells were analysed. In all analyses, CEA tetramer staining was compared with CMV tetramers as a negative control.

#### Cytokine secretion

Production of cytokines by the total population of DTH-derived cells was measured in response to T2 cells pulsed with CEA-peptide or an irrelevant HLA-A2.1 binding peptide (tyrosinase or G250). Cytokine production was measured in supernatants after 16 h by cytometric bead array (Th1/Th2 Cytokine CBA 1; BD Pharmingen, Breda, Netherlands). IFN-*γ* production was considered positive when IFN-*γ*-levels of DTH-infiltrating T cells were more than 10-fold higher after co-incubation with CEA-loaded T2 cells compared with co-incubation with T2 cells loaded with irrelevant antigen.

## Results

### Patients and toxicity

In all, seven patients were included in this study with a median age of 55 years (range 47–75). All patients had stage III colon cancer. Most patients experienced grade 1 fever and flu-like symptoms after vaccination. One patient had a grade 2 allergic reaction of unknown cause after two vaccinations, consisting of generalised exanthema with fever and pruritus for which he was treated with anti-histamines. The third vaccination was without symptoms. Other toxicities (grade 2 nausea/vomiting, one patient; grade 1 diarrhoea, one patient; grade 3 neurotoxicity, one patient) were considered to be related to the chemotherapy. With a follow-up of 10–35 months (median 18 months), all patients are recurrence-free.

### Peripheral blood subsets during treatment

**Figure 2:**
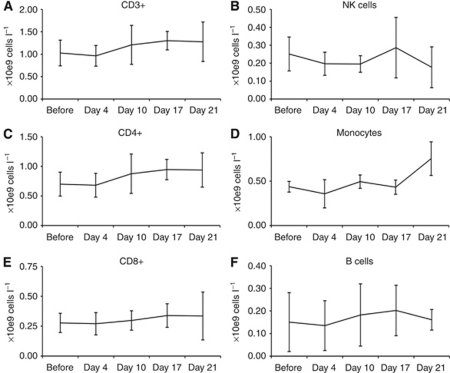
Frequencies of PBMC subsets during treatment with oxaliplatin/capecitabine and DC vaccination. Means with s.d. are depicted. (**A**) Total T cells (CD3+). (**B**) T helper cells (CD4+). (**C**) Cytotoxic T cells (CD8+). (**D**) Natural killer cells (CD3-/CD56+). (**E**) Monocytes (CD14+). (**F**) B cells (CD19+).

Peripheral blood frequencies of mononuclear cells did not significantly decrease during treatment with oxaliplatin/capecitabine (Figure 2, Supplementary Figure 1). The total number of T cells (CD3+), T helper cells (CD4+), cytotoxic T cells (CD8+), B cells (CD19+), monocytes (CD14+) and natural killer cells (CD3-/CD56+) remained stable during chemotherapy.

### Non-specific immune reactivity during oxaliplatin/capecitabine

**Figure 3:**
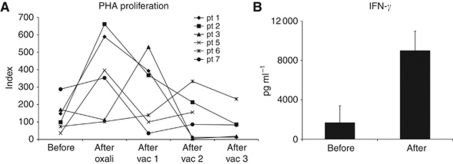
(**A**) Proliferative non-specific T-cell response upon PHA-stimulation during treatment with oxaliplatin/capecitabine and DC vaccination. In all patients, an increase in proliferation was observed during treatment. (**B**) Also the IFN-*γ* production upon PHA-stimulation increased during treatment (data of four tested patients are given; the means with s.d. are depicted).

To test whether treatment with oxaliplatin/capecitabine affects overall T-cell reactivity that could influence the efficacy of DC vaccination, we tested the PHA-induced T-cell proliferative response at different time points during treatment (Figure 3). Surprisingly, we found a striking increase in the proliferative capacity of peripheral blood T cells, in most cases directly after infusion of the oxaliplatin (Figure 3A). In addition, the IFN-*γ* release upon PHA-stimulation increased (Figure 3B). As this has not been reported before or after DC vaccination, and as we also observed this in a control group of patients that were treated with platinum compounds alone (Supplementary Patients and methods and Supplementary Figure 2), we conclude that this is a platinum-effect and not due to the DC vaccine. When peripheral blood lymphocytes of three healthy donors were stimulated with PHA after 24 h of *in vitro* culture with oxaliplatin, we did not observe an enhanced effect of the platinum treatment (Supplementary Figure 3).

### KLH-specific immune responses during oxaliplatin/capecitabine

**Figure 4:**
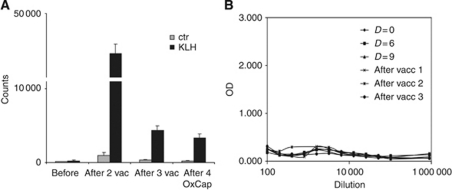
All patients showed a proliferative CD4+ T-cell response against the control antigen KLH. (**A**) A representative patient (patient 7) is shown. (**B**) However, B-cell responses against KLH upon vaccination could not be detected in 6 out of 7 patients.

To test whether *de novo* antigen-specific T- and B-cell immune responses can be induced during treatment with oxaliplatin and capecitabine, we loaded the DCs with the control antigen KLH and measured the proliferative T-cell response and antibody response (Figure 4, Table 1). We observed a robust CD4+ proliferative response against KLH in all patients, comparable to previous studies in colorectal cancer and melanoma patients who were vaccinated with the same vaccine without chemotherapy (Figure 4A) (de Vries *et al*, 2003, 2005a; Lesterhuis *et al*, 2006). However, in all but two patients antibody responses against KLH were absent (Figure 3B). In the patients with an antibody response, this response was only weak. This is in contrast to our previous studies in which we could detect a response in almost all vaccinated patients (de Vries *et al*, 2003).

### Vaccine-induced CEA-specific immune responses during oxaliplatin/capecitabine

**Figure 5:**
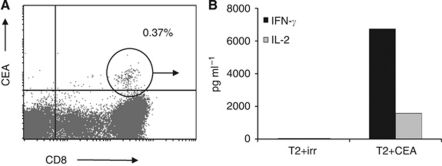
In four out of seven patients, CEA-specific DTH-infiltrating T cells were observed after completion of vaccination, either by (**A**) tetramer staining or (**B**) IFN-*γ* and IL-2 release upon co-culture with CEA-loaded target cells. Data from a representative patient (no. 2) are shown.

We next determined the vaccine-induced tumor antigen-specific immune response in DTH biopsies as previously published (de Vries *et al*, 2005a; De Vries *et al*, 2007). In four out of seven patients, we found CEA-specific T cells by means of tetramer analysis (Figure 5A, Table 1). In a previous cohort of colorectal cancer patients that were treated with the same vaccine without chemotherapy, we found CEA-specific DTH-infiltrating T cells in 5 out of 11 patients after one vaccination cycle (Lesterhuis *et al*, 2006). Thus, the efficacy of the vaccine does not seem to be negatively influenced by chemotherapy in terms of the induction of tumor antigen-specific immunity. Furthermore, the functionality of the T cells remained unaffected as they produced high amounts of IFN-*γ* and IL-2 upon co-culture with CEA-loaded target cells (Figure 5B).

## Discussion

Despite ample evidence for chemotherapy-induced immunogenic cell death, much less is known about the effect of chemotherapy on the effector lymphocytes of the immune system *in vivo* in cancer patients. Therefore, we conducted a clinical pilot trial to test the immunogenicity of a DC vaccine in stage III colon cancer patients treated with standard adjuvant oxaliplatin and capecitabine chemotherapy. We found that robust *de novo* KLH-specific T-cell responses could very well be induced during the chemotherapy regime. However, B-cell responses were hampered when compared with previous studies using the same vaccine without chemotherapy (de Vries *et al*, 2003, 2005a). No additional toxicity was observed, apart from the side effects that frequently occur for both treatment strategies separately.

In four out of seven patients, we observed a functional CEA-specific T-cell response upon vaccination with CEA-loaded DCs. Although the number of patients is limited, this is comparable to a previous cohort of colorectal cancer patients that were treated with the same vaccine without chemotherapy (Lesterhuis *et al*, 2006). To date, none of the treated patients have had a relapse. However, given the adjuvant nature of the treatment, the short follow-up and the limited numbers of patients, no conclusion can be drawn on the clinical efficacy of adding DC vaccination to standard adjuvant chemotherapy. Other studies investigating the combination of immunotherapy and chemotherapy have been performed in colorectal cancer patients. Kaufman and colleagues vaccinated metastatic colorectal cancer patients with a CEA-ALVAC vaccine in combination with irinotecan, 5-FU and leucovorin and found that the CEA-specific T-cell response was unaffected by the chemotherapy (Kaufman *et al*, 2008). Similar observations were made, using the same chemotherapeutics but different vaccines (Weihrauch *et al*, 2005; Harrop *et al*, 2008).

Surprisingly, the non-specific T-cell proliferative capacity increased upon chemotherapy treatment in most patients shortly after infusion of the oxaliplatin. Previously, others had found a similar effect for gemcitabine in a murine model (Nowak *et al*, 2002), but for platinum-based compounds this has not been reported before. Although oxaliplatin has been described to induce an immunogenic type of cancer cell death, which could result in DC activation and subsequent non-specific T-cell activation (Apetoh *et al*, 2007; Ghiringhelli *et al*, 2009), we consider this mechanism less likely in our study as the patients were treated in an adjuvant setting, that is in the absence of macroscopic tumor. A possible explanation for the increased T-cell activation may lie in our recent finding that platinum compounds dephosphorylate signal transducer and activator of transcription 6 (STAT6) (Lesterhuis *et al*, 2010). This protein is of crucial importance for the induction of T helper 2 immunity and hence for the development of B-cell responses. Inactivation of STAT6 may result in enhanced T helper 1 responsiveness and at the same time in decreased B-cell activation, as observed in this study. The fact that we did not find this effect when lymphocytes of healthy donors were stimulated with PHA after 24 h of culture in the presence of oxaliplatin, suggests that this mechanism mainly works through antigen-presenting cells and not lymphocytes. However, it is questionable whether the *in vitro* conditions properly mimic the *in vivo* situation. More research is needed to decipher the exact effects of platinum compounds on immune cells.

Our study is the first to investigate the combined use of specific vaccination with oxaliplatin-based chemotherapy. Oxaliplatin appears to be a promising drug to include in chemoimmunotherapeutic regimens, as it induces an immunogenic type of tumor cell death resulting in enhanced DC activation (Apetoh *et al*, 2007; Ghiringhelli *et al*, 2009; Tesniere *et al*, 2009). Our findings that the immune effector cells remain unaffected and that T-cell proliferative capacity following PHA stimulation even increases upon oxaliplatin treatment further support this strategy.

Although DC vaccinations frequently induce immune responses, objective clinical remissions are rare (Lesterhuis *et al*, 2008). One possible explanation is that DC vaccination may be more effective in residual microscopic disease, as it lacks a direct cytotoxic effect that is likely required in a macroscopic disease. On the other hand, cytotoxic chemotherapy also does not result in complete tumour eradication in the majority of patients with metastatic solid tumours. Therefore, the combination of DC vaccination with cytotoxic chemotherapy is an attractive strategy. Our data demonstrate the feasibility of the combination of these two strategies and provide the rationale for future studies that investigate the clinical efficacy of this strategy.

## Figures and Tables

**Table 1:** Antigen-specific immune responses

**Patient**	**1**	**2**	**3**	**4**	**5**	**6**	**7**
KLH CD4+ response	+	+	+	+	+	+	+
KLH antibody response	−	−	−	−	+	+	−
CEA-tetramers peripheral blood	−	−	−	−	−	−	−
CEA-tetramers DTH	−	+	nt	−	−	−	+
CEA-specific IFN-γ response in DTH	+	+	+	−	−	−	+

Abbreviations: CEA=carcinoembryonic antigen; DTH=delayed type hypersensitivity; IFN=interferon; KLH=keyhole limpet haemocyanin; nt=not tested.

Summary of all tested KLH and CEA-specific immune responses upon vaccination and oxaliplatin/capecitabine treatment.
