# Supplementary material for: A pilot study on the immunogenicity of dendritic cell vaccination during adjuvant oxaliplatin/capecitabine chemotherapy in colon cancer patients
Source: Br J Cancer. 2010 Oct 5;103(9):1415–21. doi: 10.1038/sj.bjc.6605935 (PMC2990614; doi:10.1038/sj.bjc.6605935)
Supplement: Supplementary Data [file 6605935x1.doc]

**Supplementary data**

**A pilot study on the immunogenicity of dendritic cell vaccination during adjuvant oxaliplatin/capecitabine chemotherapy in colon cancer patients**

W. Joost Lesterhuis^1^, I. Jolanda M. de Vries^2^, Erik A. Aarntzen^1^, Annemiek de Boer^2^, Nicole M. Scharenborg^2^, Mandy van de Rakt^2^, Dick-Johan van Spronsen^3^, Frank W. Preijers^4^, Carl G. Figdor^2^, Gosse J. Adema^2^, Cornelis J.A. Punt^1^

Departments of ^1^Medical Oncology, ^2^Tumor Immunology, ^4^Laboratory Medicine, Radboud University Nijmegen Medical Centre, Nijmegen, the Netherlands. ^3^Department of Internal Medicine, Canisius Wilhelmina Hospital Nijmegen

**Supplementary Patients and Methods**

Because we found enhanced T cell proliferation after oxaliplatin infusion, during DC vaccination, we questioned whether this could be caused by the DC vaccine or by the oxaliplatin. Furthermore, we questioned whether this could be an oxaliplatin-specific or general platinum effect. For this reason we obtained PBMCs from 2 patients with squamous cell head and neck carcinoma and 1 patient with cervical cancer that were treated with weekly cisplatin (40 mg/m^2^) in combination with local radiotherapy and tested non-specific T cell proliferative capacity by PHA stimulation. Patients gave written informed consent. Blood was obtained before and 3-4 days after infusion of cisplatin.

PBMCs were obtained by density-gradient centrifugation. Cells were plated in 96-wells U-bottom plates, 2x10^5^/well, in RPMI/human serum 5% and Phytohaemagglutinin (PHA) was added (1 μg/ml). After 24 hours supernatant was harvested for cytokine production analysis. After 3 days 1 µCi/well of 3H-thymidine was added to the culture for 8 hours, after which proliferation was stopped by storing the culture plate at -20°C. Incorporation of 3H-thymidine was measured in a ß-counter. Production of cytokines was measured in supernatants after 16 hours by cytometric bead array (Th1/Th2 Cytokine CBA 1; BD Pharmingen). Data were analyzed statistically by means of analysis of variance (ANOVA) and Student-Newman-Keuls-test. Statistical significance was defined as p < 0.05.

In a similar experiment PBMCs were obtained from three healthy voluntary donors by density-gradient centrifugation. The monocytic fraction was removed by plastic adherence during 1 hour at 37°C. Cells were plated in 96-wells U-bottom plates, 2x10^5^/well, in RPMI/human serum 5% either in the presence or absence of oxaliplatin (Eloxatin©) at a concentration of 4 μg/ml. After 24 hours the cells were washed and PHA was added (1 μg/ml). Proliferation was measured according to the above-described protocol.

**Supplementary figures**

**
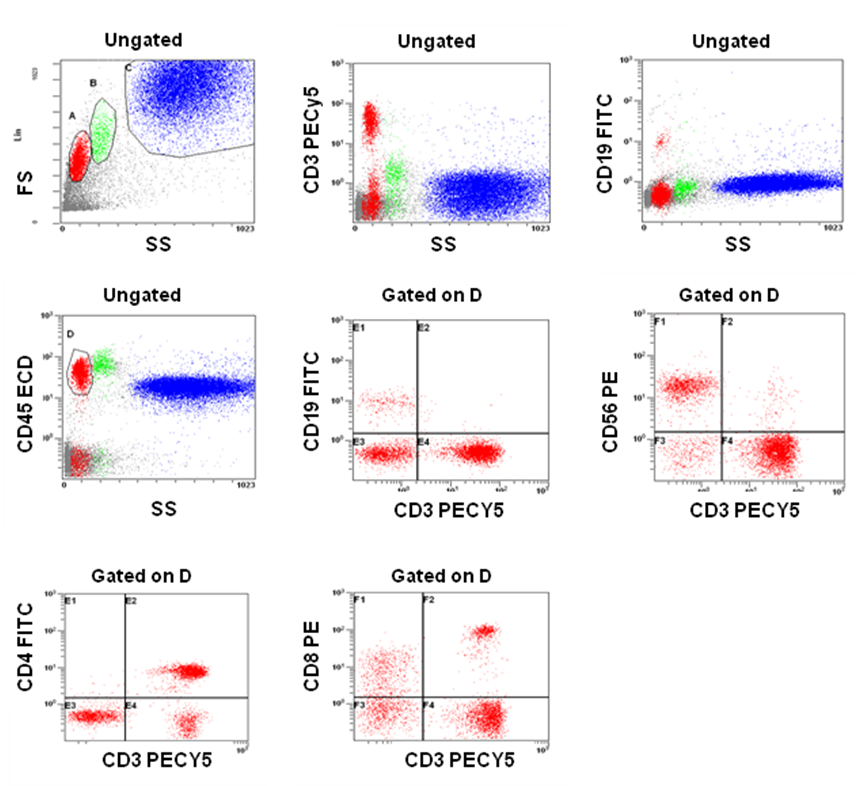
**

**Supplemental Figure 1.** Flow cytometrical determination of lymphocyte subpopulations. Gating of lymphocytes on CD45 (red) is verified by back gating from the respective marker against SS. Lymphocyte subpopulations are determined in the respective quadrants. Monocytes were determined from the CD45/SS plot (green). FS, Forward scatter; SS, side scatter

**A B C**

**
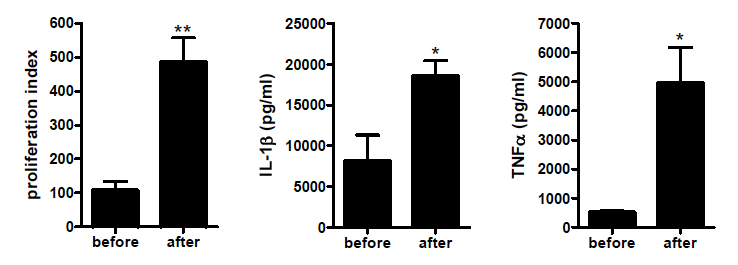
**

**Supplementary figure 2** (**A**) Proliferative response of PBMCs to phytohaemagglutinin (PHA) of cancer patients before and after treatment with cisplatin or oxaliplatin (n=5). Production of (**B**) IL-1β and (**C**) TNFα of PHA-stimulated PBMC of cancer patients before and after treatment with cisplatin (n=3; * p < 0.05, ** p < 0.01).

**
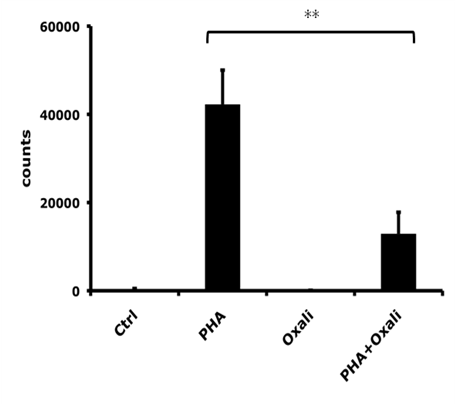
**

**Supplementary figure 3** Proliferative responses of peripheral blood lymphcytes of three healthy donors to PHA. The cells were pre-incubated for 24 hours in the presence or absence of a clinically relevant concentration of oxaliplatin (the means of three experiments with standard deviations are given; ** p < 0.01).
